# Development and validation of an inflammatory biomarkers model to predict gastric cancer prognosis: a multi-center cohort study in China

**DOI:** 10.1186/s12885-024-12483-4

**Published:** 2024-06-10

**Authors:** Shaobo Zhang, Hongxia Xu, Wei Li, Jiuwei Cui, Qingchuan Zhao, Zengqing Guo, Junqiang Chen, Qinghua Yao, Suyi Li, Ying He, Qiuge Qiao, Yongdong Feng, Hanping Shi, Chunhua Song

**Affiliations:** 1 Department of Epidemiology and Statistics, College of Public Health, Zhengzhou University, Zhengzhou, Henan 450001 China; 2 Department of Clinical Nutrition, Daping Hospital, Army Medical University (Third Military Medical University), Chongqing, 400042 China; 3 Cancer Center of the First Hospital of Jilin University, Changchun, Jilin 130021 China; 4 Department of Digestive Diseases, Xijing Hospital, Fourth Military Medical University, Xi’an, Shanxi 710032 China; 5 Department of Medical Oncology, Fujian Cancer Hospital, Fujian Medical University Cancer Hospital, Fuzhou, Fujian 350014 China; 6 Department of Gastrointestinal Surgery, First Affiliated Hospital of Guangxi Medical University, Nanning, Guangxi 530021 China; 7 Department of Integrated Traditional Chinese and Western Medicine, Zhejiang Cancer Hospital and Key Laboratory of Traditional Chinese Medicine Oncology, Zhejiang Cancer Hospital, Hangzhou, Zhejiang 310022 China; 8 Department of Nutrition and Metabolism of Oncology, Affiliated Provincial Hospital of Anhui Medical University, Hefei, Anhui 230031 China; 9 Department of Clinical Nutrition, Chongqing General Hospital, Chongqing, 400014 China; 10 Department of General Surgery, Second Hospital (East Hospital), Hebei Medical University, Shijiazhuang, Hebei 050000 China; 11 Department of Surgery, Tongji Hospital, Tongji Medical College, Huazhong University of Science and Technology, Wuhan, 430030 China; 12 Department of Gastrointestinal Surgery, Beijing Shijitan Hospital, Capital Medical University, Beijing, 100054 China; 13 Department of Clinical Nutrition, Beijing Shijitan Hospital, Capital Medical University, Beijing, 100054 China; 14 Key Laboratory of Cancer FSMP for State Market Regulation, Beijing, 100054 China; 15 Henan Key Laboratory of Tumor Epidemiology, Zhengzhou University, Zhengzhou, Henan 450001 China; 16 State Key Laboratory of Esophageal Cancer Prevention & Treatment, Zhengzhou University, Zhengzhou, Henan 450001 China

**Keywords:** Machine learning, Gastric cancer, Prognosis, Inflammatory biomarkers, Overall survival

## Abstract

**Background:**

Inflammatory factors have increasingly become a more cost-effective prognostic indicator for gastric cancer (GC). The goal of this study was to develop a prognostic score system for gastric cancer patients based on inflammatory indicators.

**Methods:**

Patients’ baseline characteristics and anthropometric measures were used as predictors, and independently screened by multiple machine learning(ML) algorithms. We constructed risk scores to predict overall survival in the training cohort and tested risk scores in the validation. The predictors selected by the model were used in multivariate Cox regression analysis and developed a nomogram to predict the individual survival of GC patients.

**Results:**

A 13-variable adaptive boost machine (ADA) model mainly comprising tumor stage and inflammation indices was selected in a wide variety of machine learning models. The ADA model performed well in predicting survival in the validation set (AUC = 0.751; 95% CI: 0.698, 0.803). Patients in the study were split into two sets – “high-risk” and “low-risk” based on 0.42, the cut-off value of the risk score. We plotted the survival curves using Kaplan-Meier analysis.

**Conclusion:**

The proposed model performed well in predicting the prognosis of GC patients and could help clinicians apply management strategies for better prognostic outcomes for patients.

**Supplementary Information:**

The online version contains supplementary material available at 10.1186/s12885-024-12483-4.

## Background

Gastric cancer (GC) is a global health problem that remains a significant contributor to the global burden of cancer [1]. The GLOBOCAN 2020 estimates of cancer incidence and mortality produced by the International Agency for Research on Cancer show that gastric cancer is the fifth most diagnosed malignancy worldwide, with more than 1 million incident cases annually and accounting for 5.6% of all cancer diagnoses [2]. Hotspots of incidence and mortality for gastric cancer exist in East Asia, Eastern Europe, and South America, with East Asia being the most affected region [3]. It is foreseeable that clinicians will encounter more and more gastric cancer cases in the future of China [4]. Due to its frequently advanced stage at diagnosis, GC has a poor prognosis [5, 6]. In China, the financial burden on families and society resulting from the treatment of gastric cancer patients is considerable, with an average cost of approximately $10,000 [7].

Conventional detections of gastric cancer include imageological examination, pathological diagnosis, and gastroscopy [8]; however, the cost of these commonly used clinical tests is typically higher. Examination, such as gastroscopy, are time-consuming and may cause minor discomfort to the patient. The interpretation of these test results is also limited by the level of the examining physician and is more subjective. The American Joint Committee on Cancer (AJCC) tumor-node-metastasis (TNM) staging system has been a vital evaluation system for guiding clinical treatment and assessing prognosis [9], which provides useful but imprecise prognostic information. However, in clinical practice, the prognosis is significantly different even in patients with the same pathological classification [10]. Consequently, there is an urgent need to establish a cost-effective model for adverse overall survival (OS) for clinicians to aggressively pursue early intervention and improve the prognosis of GC.

Tumor-related inflammation, which is regard as the 7th hallmark of tumour [11], plays a decisive role in different stages of tumor development [12]. As a result, there has been an increased focus on systemic inflammatory parameters, especially those measured by simple laboratory tests (e.g., platelet, leukocyte, neutrophil, lymphocyte, and albumin analyses). Recent studies have confirmed that many inflammatory factors, such as peripheral blood neutrophil-to-lymphocyte ratio (NLR) [13], platelet-to-lymphocyte ratio (PLR) [14], lymphocyte-to-monocyte ratio (LMR) [15] and systemic immune-inflammatory index (SII) [16], are closely associated with the prognosis of patients with gastric cancer. However, most current studies focus on the prognosis value of GC with a single inflammatory factor, and few studies have considered to establish a prognosis model by different combinations of inflammatory factors for gastric cancer. Therefore, this study attempted to establish a prognostic scoring system by combining the common inflammatory factors with the basic clinical characteristics of gastric cancer patients, and predict the survival rate of gastric cancer patients by nomograms.

## Material and method

### Study design and population

**Fig. 1:**
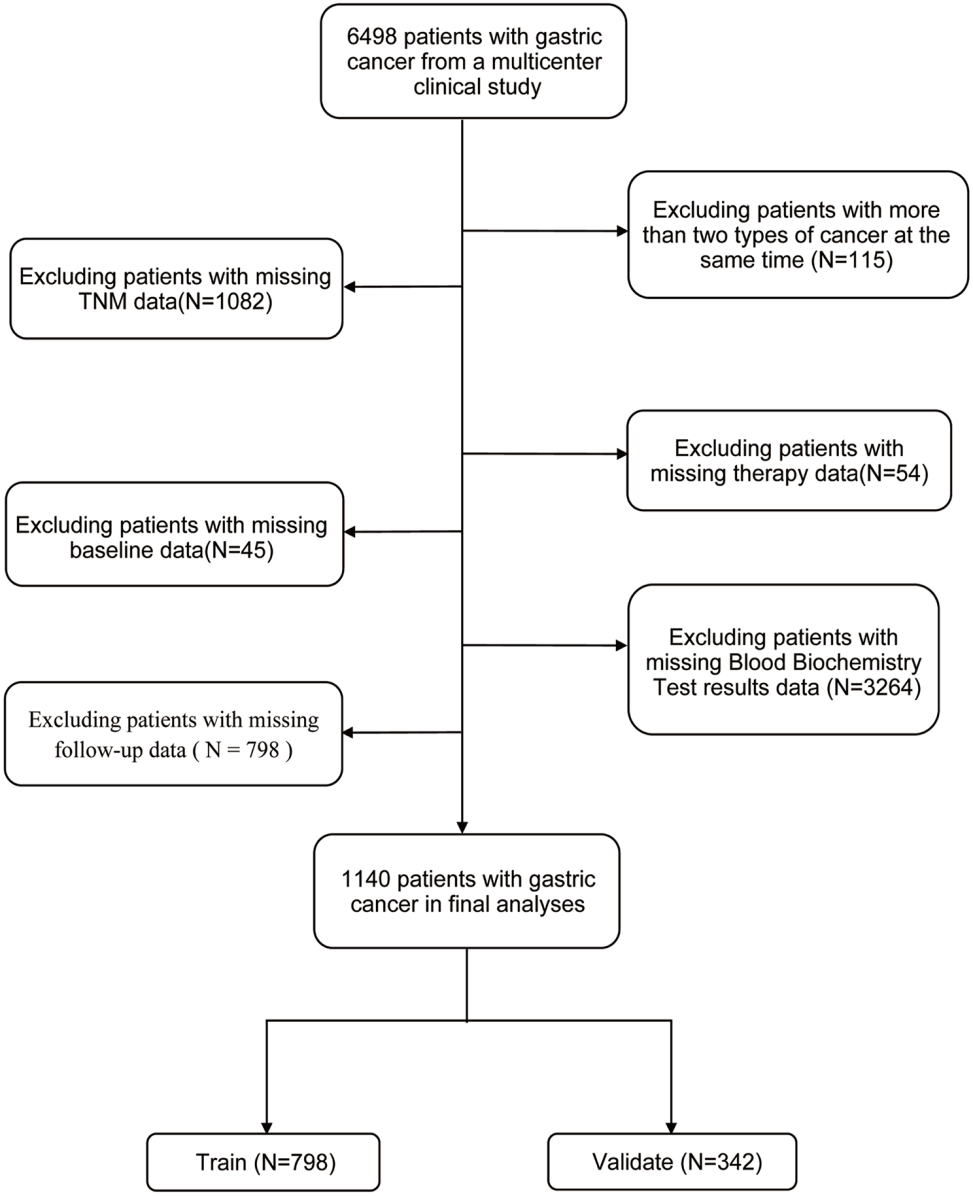
Workflow of patients inclusion

This study’s population and data were collected from a nation-wide program, the Investigation on Nutrition Status and its Clinical Outcome of Common Cancers (INSCOC). INSCOC was a multi-center retrospective cohort study conducted in China, which was registered online. It was registered in Chinese Clinical Trial Registry (ChiCTR) on December 24, 2018, with the clinical trial registration number ChiCTR1800020329. The complete protocol of this project has been described in previous study [17, 18]. The detailed inclusion and exclusion criteria can be found in Supplemental Table 1. Ultimately, 1,140 patients were included for the final analysis (Fig. 1 shows the flowchart of patients inclusion), and all of them had complete blood biochemistry test results available to support the calculation of inflammatory markers. The study was approved by the Ethics Committee of the First Affiliated Hospital of the Sun Yat-sen University. Written informed consent was obtained from all participants after explanation of the nature of the study. All data were analyzed anonymously with the removal of all identifying information. We followed the principles of the Declaration of Helsinki in this study.

### Collection of data and definition of variables

Patients’ electronic medical records were collected within 48 h of admission and interviewed by experienced medical staff using questionnaires developed in previous studies [17, 19]. These included age, gender, lifestyle (smoking; alcohol intake; tea intake), tumor-related data (tumour stage; therapy), nutritional-related metrics [the Nutrition Risk Screening 2002 (NRS 2002) score and Scored Patient-Generated Subjective Global Assessment (PG-SGA) tool], body mass index (BMI), quality of life and performance status assessment [European Organisation for Research and Treatment of Cancer Quality of Life Questionnaire-Core 30 (EORTC QLQ-C30) and the Karnofsky Performance Status (KPS)], and indicators for laboratory blood tests.

Clinical features recorded during hospitalization, including the family history, clinicopathologic staging, and blood biochemistry tests, were retrospectively collected from electronic medical records. Laboratory blood test indicators included pre-albumin levels, total protein levels, C-reactive protein levels, albumin levels, total cholesterol levels, hemoglobin levels, blood glucose levels and blood cell counts(including leukocyte, erythrocyte, lymphocyte, neutrophil and platelet). All of the blood tests were performed within 48 h of initial hospitalization, prior to antineoplastic therapy, and participants fasted for at least 9 h before blood collection. Information on lifestyle habits (including smoking, alcohol, and tea intake) was obtained through the administration of a lifestyle questionnaire. The American Joint Committee on Cancer TNM staging system (8th edition) was used for pathologic staging [9]. Supplemental Table 2 shows the formulas used to obtain the indexes required. We have established a unified endpoint determination committee for the primary study endpoints, and all study endpoints are reviewed and determined by this committee. The members of the committee were blinded to the concrete tasks of the research group.

### Outcomes

Overall survival (OS) was defined as the time from tumor diagnosis to death, loss to follow-up, or last confirmed follow-up date, which was the primary endpoint of this study. All patients were followed until June 2022.

### Machine learning models

The total population in this study was 1,140. We shuffled the data and divided the population into two sets 70% vs. 30%. We developed the model in “training ones” (*n* = 798) and used “validation ones” (*n* = 342) to estimate the model performance. We defined baseline characteristics and biochemical indices as input variables. The binary response variable was the survival status (survival or death). Several machine learning (ML) algorithms developed by current researchers were independently used to predict status of GC patients, including decision tree (DT), adaptive boost machine (ADA), random forest (RF), logistic regression (LR), support vector machine (SVM), and neural network (NNET). The algorithms were selected for their accessibility and prevalence in various cancer studies [18, 20, 21], especially gastric cancer [22, 23].

Initially, we employed a pretraining approach, utilizing all available variables to train models. The major parameters and program details for these models are indicated in Supplemental Table 3. In order to derive optimized models with fewer features and enhance the clinical application, we used the R package “Caret” for feature selection [18], which was performed with 10-fold cross-validation. In a subsequent step, the top 13 most essential factors were picked for training predigested models. The performance of the models was evaluated using receiver operating characteristic (ROC) curves. We calculated the area under the receiver operating characteristic curve (AUC) and 95% confidence interval (CI). Then we statistically compared them to identify the best machine learning algorithm. In the event that multiple algorithms exhibited comparable optimal performance, the “white box” algorithm would be selected as the preferred option, given its interpretability and ease of implementation [18]. Harrell’s concordance index (C-index), decision curve analysis (DCA), net reclassification improvement (NRI) and integrated discrimination improvement (IDI) metrics were used for model assessment to select the appropriate combination of variables.

### Statistical analysis

The predictors selected by the model were utilized for multivariate Cox regression analysis. When conducting the analysis, variables that met the Proportional Hazards Assumption were retained, and those that did not were replaced by the variables ranked 14th in the order of importance of the variables, in that order, until all 13 variables were consistent with the assumption. We calculated the cut-off values of the risk score by the Cox model, which was obtained using the R platform’s “survminer” package. Patients were divided into low-risk group and high-risk group based on the risk score. We used the Log-rank test to compare the survival rates, and Kaplan-Meier analysis was utilized to plot the survival curves. We performed univariate and multivariate analyses with Cox proportional risk models to identify independent prognostic features. We employed Hazard Ratio (HR) and 95% CI to estimate the risk of mortality in GC patients. We developed adjustment models in the multivariate adjustment model and then conducted sensitivity analysis.

Based on the results obtained from machine learning algorithms, we conducted a nomogram with chosen variables to predict the individual survival of gastric cancer patients. By comparing the survival probabilities predicted by the nomogram with the observed actual survival probabilities, we performed a calibration curve analysis with internal bootstrap correction to verify the nomogram’s discriminatory and calibration properties. A consistency index was also calculated to quantify the discriminatory performance of the nomogram. In order to facilitate more flexible applications of clinicians, we further simplified the model. We developed a nomogram using selected features on the basis and exported the best model as a Predictive Model Markup Language (PMML) file to support cross-platform deployment [18].

Continuous variables exhibited as median with superior and inferior quartiles were compared utilizing the nonparametric Wilcoxon’s rank-sum test. Categoric variables exhibited as number (%) were compared by the χ ^2^ test. We utilized the DeLong test to compare the AUC of different models. All tests were two-sided and a *P*-value of less than 0.05 was regarded as statistically significant. All analyses were implemented using the open-source software R, version 4.2.2 (The R Foundation: https://www.r-project.org/).

## Result

### Basic characteristics

We indicate extensive baseline characteristics of the patients in the Table 1. A total of 1,140 patients included in this study with a median age of 65.0 years, accounting for 798 males and 342 females. The predominant clinical stages were III (44.65%) and IV (22.90%). Family history of tumor was found in 177 patients. All participants were randomized into a training cohort (*n* = 798) and a validation cohort (*n* = 342) to further investigate the predictive value of machine learning based models in GC cancer patients. Table 1 demonstrated the comparison and revealed no significant differences in clinical and demographic characteristics between the two groups (Table 1).

**Table 1:** Baseline characteristics of the study population

Characteristics	Overall patients(*n* = 1,140)	Training cohort(*n* = 798)	Validation cohort(*n* = 342)	*P* value
gender(%)				0.632
Male	798(70.00)	562(70.43)	236(69.01)	
Female	342(30.00)	236(29.57)	106(30.99)	
Age(%)				0.990
age, <65	543(47.63)	380(47.62)	163(47.66)	
age, ≥65	597(52.37)	418(52.38)	179(52.34)	
Age	65.00(57.00-72.00)	65.00(57.00-72.00)	65.00(57.00-72.00)	0.951
BMI(%)				0.951
underweight(<18.5)	254(22.28)	170(21.30)	78(22.80)	
normal(18.5-23.9)	650(57.02)	462(57.89)	194(56.73)	
overweight(24-27.9)	207(18.16)	146(18.30)	61(17.84)	
obesity(≥28)	29(2.54)	20(2.51)	9(2.63)	
BMI	21.00(18.80-23.50)	21.00(18.90-23.53)	20.80(18.78-23.43)	0.539
Smoking, yes(%)	497(43.60)	360(45.11)	137(40.06)	0.115
Alcohol, yes(%)	265(23.25)	194(24.31)	71(20.76)	0.193
Tea, yes(%)	262(22.98)	179(22.43)	83(24.27)	0.499
Diabetes, yes(%)	83(7.28)	52(6.52)	31(9.06)	0.129
Hypertension, yes(%)	179(15.70)	124(15.54)	55(16.08)	0.817
History, yes(%)	177(15.53)	122(15.29)	55(16.08)	0.735
TNM(%)				0.119
I	134(11.75)	91(11.40)	43(12.57)	
II	236(20.70)	164(20.55)	72(21.05)	
III	509(44.65)	373(46.75)	136(39.77)	
IV	261(22.90)	170(21.30)	91(26.61)	
Therapy(%)				0.740*
Surgery	621(54.47)	444(55.64)	177(51.75)	
Chemotherapy	427(37.46)	291(36.47)	136(39.77)	
Radiotherapy	3(0.26)	2(0.25)	1(0.29)	
Chemoradiotherapy	5(0.44)	3(0.37)	2(0.59)	
Others	84(7.37)	58(7.27)	26(7.60)	
PG-SGA	7.00(4.00-10.00)	7.00(4.00-11.00)	7.00(4.00-10.00)	0.984
NRS2002	2.00(1.00-4.00)	2.00(1.00-4.00)	3.00(1.00-4.00)	0.119
KPS(%)				0.640
Cholesterol, mmol/L	4.28(3.68-5.00)	4.23(3.67-4.95)	4.38(3.72-5.04)	0.196
CRP, mg/L	3.50(2.00-10.00)	3.50(1.98-10.10)	3.30(2.07-10.00)	0.722
Blood glucose, mmol/L	5.16(4.67-5.85)	5.15(4.69-5.90)	5.21(4.69-5.90)	0.496
Hemoglobin, g/L	119.00(102.25-136.00)	119.00(102.00-136.00)	120.00(103.78-136.00)	0.727
White Blood Cell, *10^9^/L	5.73(4.50-7.21)	5.70(4.48-7.22)	5.81(4.55-7.22)	0.483
Neutrophil, *10^9^/L	3.19(2.11-4.67)	3.16(2.10-4.64)	3.29(2.22-4.91)	0.093
Lymphocyte, *10^9^/L	1.46(1.00-1.90)	1.43(1.00-1.87)	1.50(1.03-1.90)	0.318
Red Blood Cell, *10^9^/L	4.16(3.67-4.65)	4.16(3.68-4.64)	4.15(3.67-4.65)	0.838
Platelet, *10^9^/L	223.00(168.00-283.00)	223.00(164.00-283.00)	224.00(172.75-285.10)	0.493
NLR	2.16(1.42-3.57)	2.17(1.42-3.45)	2.14(1.40-3.87)	0.725
PLR	154.35(109.73-237.24)	156.68(108.24-241.15)	150.94(110.98-223.08)	0.470
GLR	3.64(2.71-5.53)	3.65(2.73-5.64)	3.59(2.68-5.32)	0.296
ALI	38.75(22.60-60.37)	38.76(23.02-61.17)	38.74(20.99-60.15)	0.657
SII	486.72(273.87-862.13)	484.70(272.98-839.79)	503.26(274.37-875.18)	0.465
CAR	0.09(0.05-0.28)	0.09(0.04-0.29)	0.09(0.05-0.26)	0.796
Nutritional Risk Index	84.83(79.01-91.12)	84.82(78.99-91.13)	84.86(79.02-91.04)	0.755
AGR	1.43(1.22-1.67)	1.45(1.23-1.68)	1.39(1.20-1.64)	0.040
PGR	7.08(5.15-9.13)	7.11(5.19-9.22)	7.01(5.11-8.92)	0.353
PNI	46.63(42.11-50.89)	46.53(42.14-50.65)	46.80(42.10-51.30)	0.537
LCR	3440.97(1143.21-7053.73)	3377.93(1075.58-7058.65)	3510.31(1320.75-7073.92)	
mGPS(%)				0.656
Score 0	862(75.61)	598(74.94)	264(77.20)	
Score 1	159(13.95)	116(14.53)	43(12.57)	
Score 2	119(10.44)	84(10.53)	35(10.23)	
mGPS	0.00(0.00-0.00)	0.00(0.00-1.00)	0.00(0.00-0.00)	0.456
LCS(%)				0.885
Score 0	264(23.16)	184(23.06)	80(23.39)	
Score 1	724(63.51)	505(63.28)	219(64.04)	
Score 2	152(13.33)	109(13.66)	43(12.57)	
LCS	1.00(1.00-1.00)	1.00(1.00-1.00)	1.00(1.00-1.00)	0.724
CONUT(%)				0.195*
0-1	185(16.20)	5(0.63)	1(0.29)	
2-4	686(60.20)	424(53.13)	205(59.94)	
5-8	260(22.80)	340(42.61)	126(36.84)	
9-12	9(0.80)	29(6.63)	10(2.93)	
CONUT	4.00(3.00-6.00)	4.00(3.00-6.00)	4.00(3.00-5.00)	0.055

### Feature selection and Model Development

**Fig. 2:**
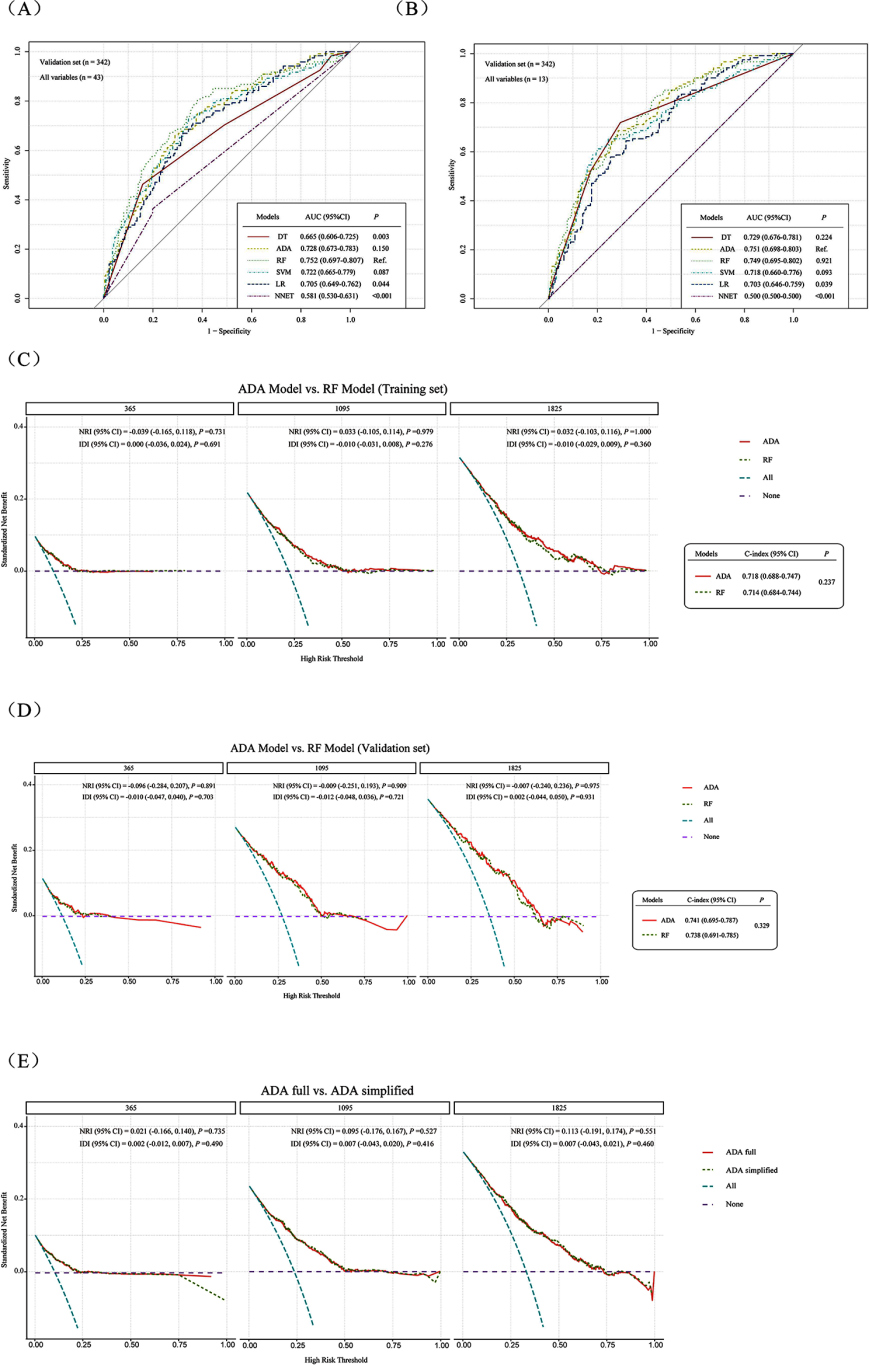
Model performance in the data (AUCs were compared using DeLong’s test). (**A**) Performance of the full ML models. (**B**) Performance of the simplified ML models after feature selection. (**C**) Comparison of the ADA and RF in training set. (**D**) Comparison of the ADA and RF in validation set. (**E**) Comparison of the full ADA and simplified ADA

We severally trained models in the training cohort by defining all attainable baseline features as the input variables and regarding survival status as the response variable. The complete models were consequently estimated in the validation cohort. The estimation results are illustrated in Fig. 2A, the RF model performed excellent with an AUC of 0.752 (95% CI: 0.697, 0.807). Setting the RF model as the reference, we statistically compared the models’ efficiency. The performance of ADA and SVM were similar with the RF (*P* = 0.150; *P* = 0.087), while the other models displayed significantly inferior efficiency (*P* < 0.05).

The Caret framework has an internal function to appraise variables’ importance; thus, we calculated our models with the function. DT, SVM, and ADA used the AUC as the model metric, different from those models(LR, t statistic; RF, mean decrease accuracy; NNET, connection weights). Supplemental Fig. 1 depicts the comparative importance of the input variables of the complete models. The input variables for each model were ranked in order of importance, and the top 13 variables (top 30%; Supplementary Table 4) for each model were selected for model reconstruction. In the validation sets, the RF model still performed better with an AUC of 0.763 (95% CI: 0.711, 0.815). The RF was set as the reference model; the ADA (*P* = 0.137) and DT (*P* = 0.099) were statistically comparable with the RF, while other models displayed inferior performance (*P* < 0.05) (Supplemental Fig. 2).

Since the purpose of this study was to assess the prognosis of gastric cancer using inflammatory indicators, the variables in the simplified model were evaluated separately for the Proportional Hazards Assumption (PH Assumption) before building the Cox regression model. LCR (*P* < 0.0001), LCS (*P* = 0.007), GLR (*P* = 0.021), and PLR (*P* = 0.012) did not satisfy the PH Assumption. After the replacement, the variables in the new simplified model were all consistent with the PH Assumption, and we listed the 13 variables utilized to construct the novelty model in Supplemental Table 5.

In the retrained streamlined models, ADA model displayed the most excellent performance, with an AUC of 0.751 (95% CI: 0.698, 0.803). The DT (*P* = 0.224), SVM (*P* = 0.093), and RF (*P* = 0.921) models were statistically comparable with the ADA, while other streamlined models performed worse (*P* < 0.05) (Fig. 2B). We evaluated the influence of selecting feature on the efficiency of diff ML algorithms (Table 2). Reducing the number of input variables from 43 to 13 strikingly decreased the performance of DT and NNET (all *P* < 0.05), while the effects on ADA, RF, SVM, and LR were not significant (all *P* > 0.05).

**Table 2:** Comparison of the model performance before and after feature selection

	Full models (43 variables)	Simplified models(13 variables)	*P*
Decision Tree	0.665(0.606–0.725)	0.729(0.676–0.781)	0.0032
Adaptive boost machine	0.728(0.673–0.783)	0.751(0.698–0.803)	0.1861
Random Forest	0.752(0.697–0.807)	0.749(0.695–0.802)	0.8683
Support vector machine	0.722(0.665–0.779)	0.718(0.660–0.776)	0.8626
Logistic Regression	0.705(0.649–0.762)	0.703(0.646–0.759)	0.8155
Neural network	0.581(0.530–0.631)	0.500(0.500–0.500)	0.0017

We compared the better-performing ADA and RF models, one of them for future use. The combinations of variables in the ADA and RF models were incorporated into the Cox regression models separately. The final model was selected by comparing the calculated C-index, IDI, NRI, and DCA curves in the training and validation datasets (Fig. 2C and D). The ADA model had a higher C-index value than the RF model in the both training and validation sets, but the differences were not statistically significant. The NRI and IDI metrics displayed that the ADA rarely improved regarding discrimination compared with the RF. Observed the curve of DCA, we found the ADA and RF model displayed comparable performance when the threshold probability for predicting 1-, or 3-, 5-year survival in GC patients was > 0.05 or 0.10. Clinicians who utilized either model to predict the probability of survival could gain more benefits than those who chose the strategy of treating all patients or none patients. The ADA displayed lightly better performance than the RF in partial intervals. Therefore, we selected the ADA for future use.

Subsequently, we made a comparison between the complete ADA model with 43 variables and the simplified ADA model with 13 variables in terms of their clinical usefulness and discrimination (Fig. 2E). By comparing the metrics of NRI and IDI, we observed that the complete model barely improved regarding discrimination compared to the streamlined model. Although the differences between the two models were out of significance, the DCA curve displayed that using either the ADA or the streamlined model still gained more benefits in predicting the probability of survival.

### Validation of the Survival Prediction ability of Prognostic Model

The predictors in the simplified ADA model were included in the final prognostic Cox model. Supplemental Table 6 displays the HRs (95% CIs) and *P*-value of the factors in the prognostic model. The complete equation of the prognostic model is demonstrated in Supplemental Table 6’s footnote. According to the calculated cut off score of the risk classification (0.75), the participants were split into two groups–“high-risk ones” and “low-risk ones” (Supplemental Table 7), and the Kaplan-Meier analysis was used to plot a survival curve (Supplemental Fig. 3A-3B). The sensitivity analyses indicated a consistent result (Supplemental Table 8). Furthermore, the results of the ROC curve demonstrated better performance for 1-, 3-, and 5-year both in the training and validation datasets (Supplemental Fig. 3C-3D). The calibration curve’s plot displayed excellent consistency between the observed actual probability and the OS predicted by the prognostic model in the validation set. Next, we performed 1,000 internal cross-validations of this model using the bootstrap method. Supplemental Fig. 3E showed the C-index of 1-, 3-, 5-year.

### Nomogram Model for clinicians after Simplification

**Fig. 3:**
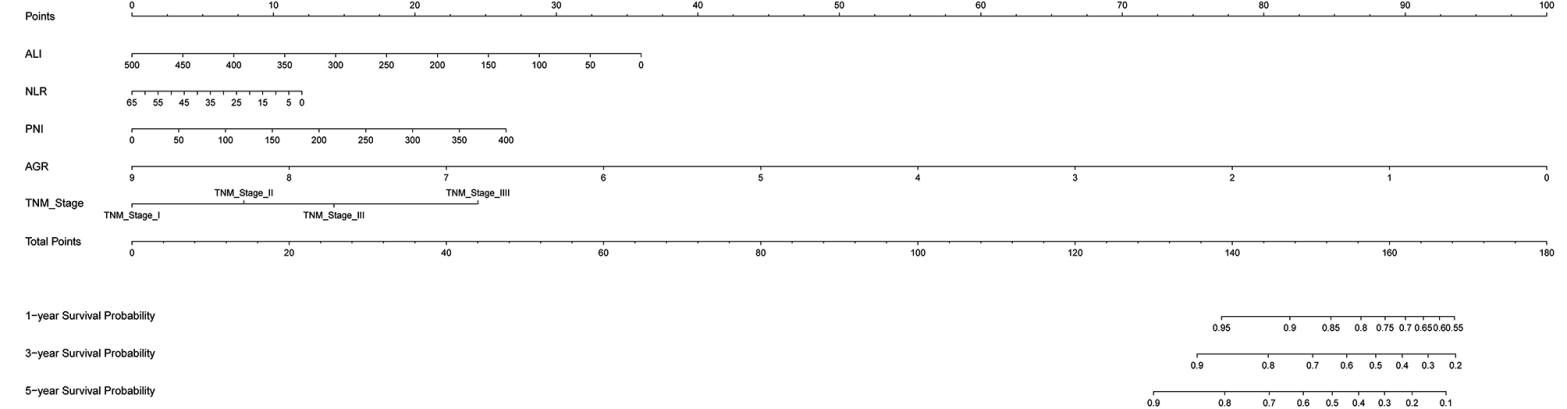
The nomogram for overall survival prediction in GC patients

We screened out independent predictors on the very borderline of significance in the multivariate Cox analysis, including TNM(*P* < 0.001), ALI(*P* = 0.009), and AGR(*P* = 0.058). Through literature review, we found that NLR and PNI, two inflammatory indicators, are also commonly used to predict the prognosis of patients with gastric cancer. Combined with this study, we found that NLR and PNI also had relatively small *P* values (*P* < 0.2) in the multivariate Cox analysis. After completing the two-step simplification process, we reduced the indicators involved in establishing the prognosis model to TNM, ALI, AGR, NLR, and PNI. After further simplification of the prognostic model, we screened for five independent predictors. The final nomogram model incorporated these five predictors to predict patient survival (Fig. 3). The complete equation of the prognostic model is displayed in Table 3’s footnote. The OS of GC patients was positively related to tumor stage II, III, and IV (all HRs > 1, all *P* < 0.05). However, it was negatively related to the AGR and the ALI (all HRs < 1, all *P* < 0.05).

**Table 3:** Cox analysis results of Nomogram Model

Characteristics	HR (95%CI)	*P*
TNM		
I	Reference (HR = 1)	
II	1.984 (1.426, 2.541)	0.016
III	3.317 (2.801, 3.833)	< 0.001
IV	8.303 (7.782, 8.824)	< 0.001
AGR	0.643 (0.475, 0.811)	< 0.001
ALI	0.994 (0.990, 0.998)	0.003
NLR	0.985 (0.960, 1.009)	0.213
PNI	1.001 (0.995, 1.007)	0.709

*Notes* Risk score of the Cox model: 0.6849 × tumor stage II (yes = 1, no = 0) + 1.1991 × tumor stage III (yes = 1, no = 0) + 2.1166 × tumor stage IV (yes = 1, no = 0) − 0.4418 × AGR − 0.0059 × ALI − 0.0156 × NLR + 0.0012 × PNI

**Fig. 4:**
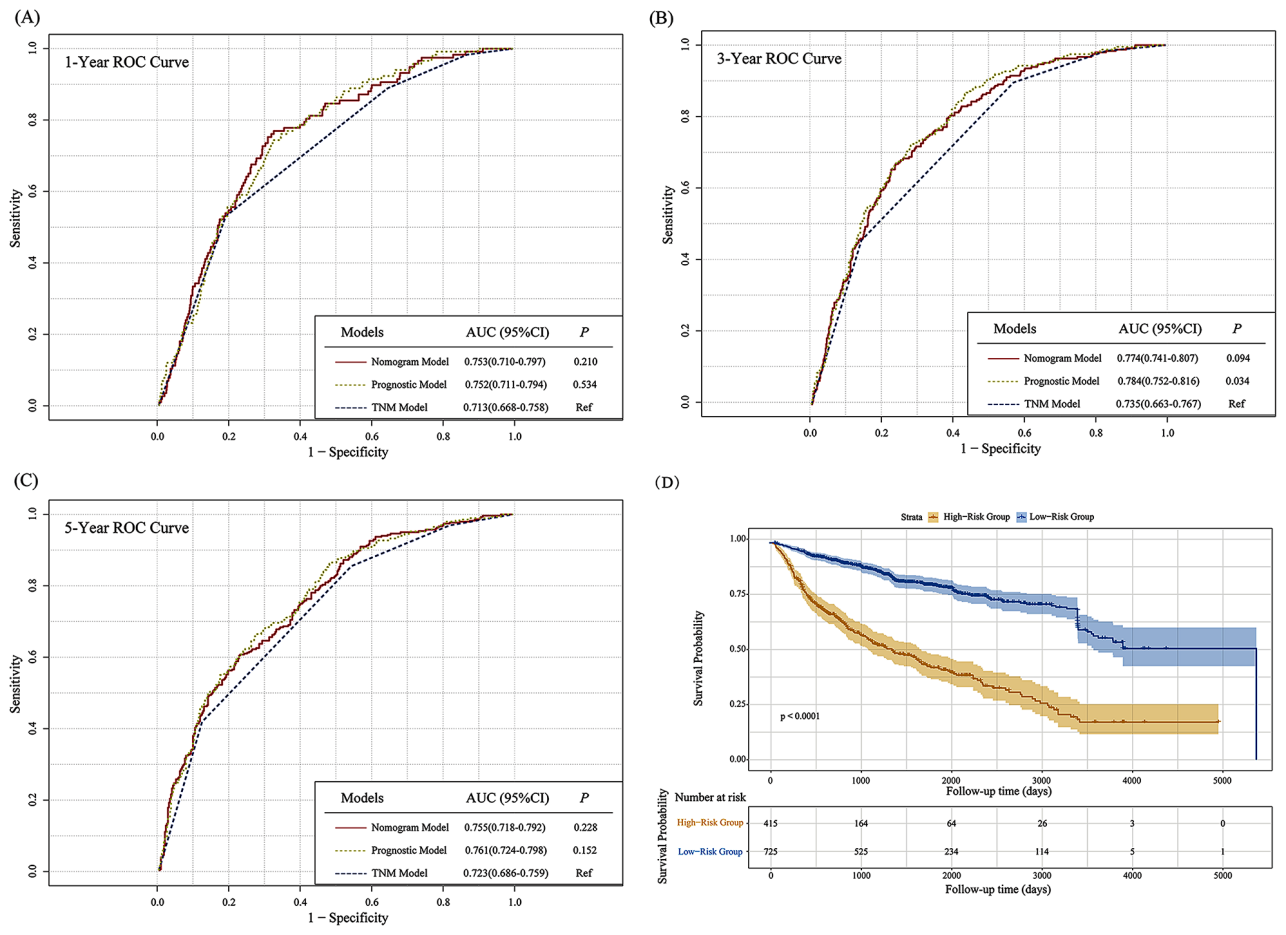
Nomogram Model performance in total patients (AUCs were compared using DeLong’s test). (**A**) Comparison of 1-year prognostic ROC for Risk Score calculated by Nomogram Model, Cox Model and TNM Model in total patients. (**B**) Comparison of 3-year prognostic ROC for Risk Score calculated by Nomogram Model, Cox Model and TNM Model in total patients. (**C**) Comparison of 5-year prognostic ROC for Risk Score calculated by Nomogram Model, Cox Model and TNM Model in total patients. (**D**) The Kaplan-Meier survival curves of Risk Score calculated by Cox Model in total patients

The Nomogram model showed better performance than the classical American Joint Committee on Cancer TNM staging system (*P* = 0.094), with AUC values of 0.753, 0.774, 0.755 at 1, 3, 5 years (Fig. 4A and C). In the subgroups of the population using different treatment modalities, the column-line diagram model also outperformed the classical TNM staging system (Supplemental Figs. 4–6). In particular, for the population receiving surgical treatment and chemotherapy, the AUC value of the column-line diagram was higher than that of the TNM system in predicting the 3-year prognosis (*P* = 0.121; *P* = 0.200), with AUC values of 0.769 (Supplemental Fig. 4B) and 0.726 (Supplemental Fig. 5B). We performed predictive analysis on the established risk score of the Nomogram model, and survival analyses showed that the cohort’s high-risk group had a significantly lower level of OS than the low-risk group (Table 4). The sensitivity analysis showed a similar result (Supplemental Table 9). Patients were stratified based on the risk score cut-off value (0.42), and subsequently, Kaplan-Meier survival curves were generated (Fig. 4D). The Cox model was consequently applied as a web-based risk calculator (https://gcnomogram2023.shinyapps.io/dynnomapp/), likewise an offline risk calculation nomogram.

**Table 4:** The univariate and multivariate analysis of risk score in total patients

Variables	OS (model 0)			OS (model 1)			OS (model 2)	
	Crude HR (95%CI)	Crude *P*		Adjusted HR (95%CI)	Adjusted *P*		Adjusted HR (95%CI)	Adjusted *P*
Total patients								
Risk Score	2.718 (2.363–3.126)	< 0.001		2.751 (2.385–3.172)	< 0.001		2.646 (1.842-3.800)	< 0.001
Low risk group	Reference (HR = 1)			Reference (HR = 1)			Reference (HR = 1)	
High risk group	3.820 (3.115–4.463)	< 0.001		3.904 (3.171–4.807)	< 0.001		1.470 (1.254–1.724)	< 0.001

Model 0: non-adjustment model

Model 1:adjusted for age, gender, alcohol, history

Model 2: adjusted for age, gender, TNM, BMI, smoking, alcohol, KPS, therapy, diabetes, hypertension, history, PG-SGA, NRS2002, EORTC QLQ-C30

## Discussion

This study was part of a prospective multi-center cohort study that included patients with gastric cancer from several regions in China. The study aims to establish a new inflammation-related score system that can more precisely predict the OS of gastric cancer patients. We tackle this challenging problem with the machine learning approach based on inflammatory indicators and traditional clinical characteristics. This study may help clinicians decide how to treat high-risk patients and guide them in developing management strategies to improve patient outcomes.

Gastric cancer is a malignant tumor characterized by high morbidity and mortality, which has been widely reported in East Asia [3]. TNM staging, as an important evaluation system for guiding clinical treatment and assessing prognosis, are not yet adequate for the needs of individualized and accurate treatment for GC patients [22]. Some studies have demonstrated that the prognostic model constructed by machine learning method and Cox regression analysis has a significantly better evaluation performance than TNM staging [24–26]. Therefore, the selection of more representative and easier-to-access metrics for constructing model to accurately appraise the prognosis of gastric cancer patients is a major concern for researchers.

In the prognostic model constructed by the machine learning method, the prognosis of gastric cancer patients was closely related to intermediate and advanced cancer. Inflammatory factors were ranked high in relative importance for the diagnosis of malignant disease. The consequences of multifactorial Cox analysis displayed that AGR, ALI and TNM stage were independent prognostic indicators for GC patients. Several studies have shown that reduced pretreatment ALI is an independent risk factor for OS in cancer patients, particularly GC patients [27–30]. These findings are consistent with this research. The results of Nomogram Model in this study showed that the HR of ALI was 0.994, indicating that high levels of ALI have a protective effect on the prognosis of patients with GC. AGR was deemed to be a valid combination of the two predictive indices. Previous meta-analyses have shown that a lower level of AGR is related to lower survival rates in digestive system cancers [31]. The investigators concluded that AGR can be a valid prognostic indicator for GC, which may help clinicians find GC patients with high-risk who require pre-treatment interventions in their clinical practice [32–34]. In this study, the *P* < 0.05 of AGR after multifactorial Cox analysis, and the HR was 0.643. Combined with previous studies, AGR can be considered as a protective factor for GC prognosis. Previous studies have indicated that the remaining inflammatory factors such as NLR and PNI can also be utilized as independent prognostic factors in gastric cancer patients [35–37]. However, since this study included them as continuous variables in the multifactorial Cox analysis, the results indicated that the P-values all exceeded 0.05, and further studies can be conducted after finding the appropriate cut-off values to classify them in subsequent studies.

Despite the extensive global adoption of the TNM stage system, it still has some limitations. Until now, TNM staging has always been evaluated on the basis of anatomical factors. However, tumor diagnosis and treatment have entered the era of precision therapy, and there is a particular need to incorporate markers related to inflammatory response into the prognostic assessment [38, 39]. To alleviate the perceived limitations, several researchers have derived new tools or nomograms [40, 41]. Many studies have correlated systemic inflammation with the development and progression of malignancy and patient prognosis [42, 43]. The tumor inflammatory microenvironment is complex and dynamic, involving crosstalk between various immune cells and tumor cells [44]. This phenomenon not only promotes the development and progression of cancer, but also significantly affects patient prognosis [45]. Compared to other assays, blood biochemical tests are easier and more convenient, and their price is relatively low; therefore, inflammatory factor indices obtained by this method are gradually coming into the limelight. Inflammatory prognostic scores calculated using inflammatory factors and related parameters have shown promise in a variety of tumor types. Accordingly, the nomogram based on the systemic immune and inflammation indicator is superior to the available systems for predicting survival in patients with gastric cancer. Facing multiple metrics, how to choose the right combination of metrics to build a model is also an essential conundrum. To solve this problem, ML algorithms have become the first-line [46–48]. Several studies have exhibited the efficacy of machine learning algorithms in selecting indicators to construct predictive models [24, 25, 49]. Turkki R [50] developed support vector machines and artificial neural networks to predict breast cancer prognosis and obtained good efficiency of the model. Wang et al. used five classifiers [51] to sort the postoperative clinical characteristics of colon cancer patients by importance. In this way, we can develop survival prediction models suitable for the survival prediction of gastric cancer patients in China.

A distinguishing characteristic of the prognostic model used in this study is the incorporation of various inflammatory factors and the associated inflammatory prognostic scores. We selected variables from the best-performing models in the machine learning method as predictors to construct the column line graph model and used the training set to predict OS in GC patients. 1,000 replicate bootstraps showed good model accuracy as evidenced by a C-index value of 0.724. Compared with TNM Model, 1, 3 and 5-year OS AUC values indicated the high diagnostic effectiveness of our model in predicting OS in GC patients. In order to mitigate the potential confounding effects of diverse treatment modalities on the prognosis of patients with GC, we also compared the performance of our Nomogram Model and the TNM Model within subgroups receiving different treatments. The findings revealed that our Nomogram Model outperformed the traditional TNM staging system, with significantly higher AUC values in predicting 3- and 5-year prognosis, particularly among patients undergoing surgery and chemotherapy. The simplified Nomogram model, which was facilitated to be easier for clinicians to use, also showed superior performance compared to the TNM model. We separated patients into high and low groups by the risk score calculated simplification model and found that this risk-based stratification also significantly differentiated patient OS.

The primary strength of this study lies in its prospective multicenter design, which employs multiple machine learning approaches to screen metrics and compare/validate the constructed models. The metrics used to build the Nomogram Model are readily available in clinical practice and offer greater convenience for stratifying clinical prognosis and optimizing treatment strategies. Currently, patients with gastric cancer are more likely to undergo surgery or chemotherapy, and our constructed Nomogram Model demonstrates significant superiority over TNM staging in these two groups, warranting its clinical application.

There are several potential limitations in this study. Firstly, because of the study’s retrospective design, the sample may suffer from selection bias. Besides, the prognosis of the gastric cancer patients is intricate and vulnerable to physical and environmental elements. Other confounding elements that may influence patients’ prognoses need to be considered. Third, despite the DCA results supporting the clinical utility of the final model, we still need more assessment of patients under treatment-specific data to prove our consequence. Fourthly, among the 1,140 participants included in the study, only 7 received immunotherapy. Due to the small number of participants, the analysis was not possible, the subgroup analysis of the prognosis and efficacy of immunotherapy patients was not supplemented in this study. In addition, the inflammatory indices and their dynamic changes with treatment response also need to be explored. However, it is regrettable that we do not currently gather such data. We will consider incorporating this section if additional data becomes available in the future. Finally, although we internally validated the predictive value of our model, the results were not proved by other independent datasets, thus we could not confirm the external validity. Future studies with larger sample sizes and broader clinical characteristics of gastric cancer patients are warranted to address these issues.

## Conclusion

In summary, it is effective that establish prognosis model developed by inflammatory indicators for gastric cancer patients. In this research, we developed a clinical prognostic model for gastric cancer using the machine learning approach, which performed well in predicting the prognoses of gastric cancer patients combining conventional clinical features and inflammatory indicators. The model was implemented as an online tool and nomogram, which can help clinicians make decisions and guide management strategies for better prognostic outcomes for patients.

### Electronic supplementary material

Below is the link to the electronic supplementary material.

Supplementary Material 1

## Data Availability

The raw data supporting the conclusions of this article will be made available by the authors, without undue reservation.

## Abbreviations

ADA: adaptive boost machine
DT: decision tree (conditional inference tree)
IDI: integrated discrimination improvement
LR: logistic regression
ML: machine learning
NNET: neural network
NRI: net reclassification improvement
RF: random forest
SVM: support vector machine
BMI: Body Mass Index
TNM: Tumor Node Metastasis
PG-SGA: Patient-Generated Subjective Global Assessment
NRS2002: Nutrition Risk Screening
KPS: Karnofsky Performance Status
EORTC: The European Organisation for Reasearch and Treatment of Cancer
QLQC30: Quality of Life Questionnarie-Core 30
ALI: Advanced lung cancer inflammation index
SII: Systemic Immune-Inflammation Index
PLR: Platelet-Lymphocyte Ratio
NLR: Neutrophil-Lymphocyte Ratio
PNI: Prognostic Nutritional Index
CAR: C-reactive protein-Albumin Ratio
GLR: Glucose-to-Lymphocyte Ratio
LCR: Lymphocyte/C-reactive protein Ratio
AGR: Albumin-to-Globulin Ratio
PGR: Prealbumin-to-Globulin Ratio
mGPS: Modified Glasgow Prognostic Score
LCS: Lymphocyte C-reactive protein Score
CONUT Score: Controlling Nutritional Status Score
HR: Hazard ratio
CI: Confidence Interval

## References

[1] Amin MB, Greene FL, Edge SB, Compton CC, Gershenwald JE, Brookland RK, Meyer L, Gress DM, Byrd DR, Winchester DP (2017). The Eighth Edition AJCC Cancer staging Manual: continuing to build a bridge from a population-based to a more personalized approach to cancer staging. CA Cancer J Clin.

[2] Sung H, Ferlay J, Siegel RL, Laversanne M, Soerjomataram I, Jemal A, Bray F (2021). Global Cancer statistics 2020: GLOBOCAN estimates of incidence and Mortality Worldwide for 36 cancers in 185 countries. CA Cancer J Clin.

[3] Smyth EC, Nilsson M, Grabsch HI, van Grieken NC, Lordick F (2020). Gastric cancer. Lancet.

[4] Karimi P, Islami F, Anandasabapathy S, Freedman ND, Kamangar F (2014). Gastric cancer: descriptive epidemiology, risk factors, screening, and prevention. Cancer Epidemiol Biomarkers Prev.

[5] Thrift AP, El-Serag HB (2020). Burden of gastric Cancer. Clin Gastroenterol Hepatol.

[6] Wong MCS, Huang J, Chan PSF, Choi P, Lao XQ, Chan SM, Teoh A, Liang P (2021). Global incidence and mortality of gastric Cancer, 1980–2018. JAMA Netw Open.

[7] Bray F, Ferlay J, Soerjomataram I, Siegel RL, Torre LA, Jemal A (2018). Global cancer statistics 2018: GLOBOCAN estimates of incidence and mortality worldwide for 36 cancers in 185 countries. CA Cancer J Clin.

[8] Guo L, Wang Q, Chen K, Liu HP, Chen X (2021). Prognostic value of combination of inflammatory and tumor markers in Resectable Gastric Cancer. J Gastrointest Surg.

[9] Mullaney PJ, Wadley MS, Hyde C, Wyatt J, Lawrence G, Hallissey MT, Fielding JW (2002). Appraisal of compliance with the UICC/AJCC staging system in the staging of gastric cancer. Union Internacional Contra La Cancrum/American Joint Committee on Cancer. Br J Surg.

[10] Gao X, Pan Y, Han W, Hu C, Wang C, Chen L, Guo Y, Shi Y, Pan Y, Xie H (2021). Association of systemic inflammation and body mass index with survival in patients with resectable gastric or gastroesophageal junction adenocarcinomas. Cancer Biol Med.

[11] Hanahan D, Weinberg RA (2011). Hallmarks of cancer: the next generation. Cell.

[12] Fu H, Li B, Liang Z (2022). Effect of enteral immunonutrition compared with enteral nutrition on surgical wound infection, immune and inflammatory factors, serum proteins, and cellular immunity in subjects with gastric cancer undergoing a total gastrectomy: a meta-analysis. Int Wound J.

[13] Wang W, Tong Y, Sun S, Tan Y, Shan Z, Sun F, Jiang C, Zhu Y, Zhang J (2022). Predictive value of NLR and PLR in response to preoperative chemotherapy and prognosis in locally advanced gastric cancer. Front Oncol.

[14] Wang SB, Chen JY, Xu C, Cao WG, Cai R, Cao L, Cai G (2022). Evaluation of systemic inflammatory and nutritional indexes in locally advanced gastric cancer treated with adjuvant chemoradiotherapy after D2 dissection. Front Oncol.

[15] Li Z, Li S, Ying X, Zhang L, Shan F, Jia Y, Ji J (2020). The clinical value and usage of inflammatory and nutritional markers in survival prediction for gastric cancer patients with neoadjuvant chemotherapy and D2 lymphadenectomy. Gastric Cancer.

[16] He K, Si L, Pan X, Sun L, Wang Y, Lu J, Wang X (2022). Preoperative systemic Immune-inflammation index (SII) as a Superior Predictor of Long-Term Survival Outcome in patients with stage I-II gastric Cancer after radical surgery. Front Oncol.

[17] Xu H, Song C, Yin L, Wang C, Fu Z, Guo Z, Lin Y, Shi Y, Hu W, Ba Y (2022). Extension protocol for the investigation on Nutrition Status and clinical outcome of patients with common cancers in China (INSCOC) study: 2021 update. Precision Nutr.

[18] Yin L, Cui J, Lin X, Li N, Fan Y, Zhang L, Liu J, Chong F, Wang C, Liang T et al. Identifying cancer cachexia in patients without weight loss information: machine learning approaches to address a real-world challenge. Am J Clin Nutr 2022. 10.1093/ajcn/nqac251 36095136

[19] Zhou M, Xu H, Cui J, Wang K, Weng M, Guo Z, Yao Q, Zhou F, Liu M, Zhou C (2023). Variation trends of malnutrition status among malignancy inpatients in China from 2014 to 2021. Precision Nutr.

[20] Xu H, Song C, Wang C, Fu Z, Guo Z, Lin Y, Shi Y, Hu W, Ba Y, Li S et al. Investigation on nutrition status and clinical outcome of patients with common cancers in Chinese patients: a multicenter prospective study protocol. Int J Clin Trials 2020, 7.

[21] Yin L, Liu J, Lin X, Li N, Guo J, Fan Y, Zhang L, Shi M, Zhang H, Chen X (2021). Nutritional features-based clustering analysis as a feasible approach for early identification of malnutrition in patients with cancer. Eur J Clin Nutr.

[22] Liu D, Wang X, Li L, Jiang Q, Li X, Liu M, Wang W, Shi E, Zhang C, Wang Y (2022). Machine learning-based model for the prognosis of postoperative gastric Cancer. Cancer Manag Res.

[23] Xu C, Wang J, Zheng T, Cao Y, Ye F (2022). Prediction of prognosis and survival of patients with gastric cancer by a weighted improved random forest model: an application of machine learning in medicine. Arch Med Sci.

[24] Cheong JH, Wang SC, Park S, Porembka MR, Christie AL, Kim H, Kim HS, Zhu H, Hyung WJ, Noh SH (2022). Development and validation of a prognostic and predictive 32-gene signature for gastric cancer. Nat Commun.

[25] Kuntz S, Krieghoff-Henning E, Kather JN, Jutzi T, Höhn J, Kiehl L, Hekler A, Alwers E, von Kalle C, Fröhling S (2021). Gastrointestinal cancer classification and prognostication from histology using deep learning: systematic review. Eur J Cancer.

[26] Hao D, Li Q, Feng QX, Qi L, Liu XS, Arefan D, Zhang YD, Wu S (2022). SurvivalCNN: a deep learning-based method for gastric cancer survival prediction using radiological imaging data and clinicopathological variables. Artif Intell Med.

[27] Zhang X, Wang D, Sun T, Li W, Dang C (2022). Advanced lung cancer inflammation index (ALI) predicts prognosis of patients with gastric cancer after surgical resection. BMC Cancer.

[28] Chen H, Zhang F, Luo D, Guo J, Lin Y, Chen S, Yin S, Chen X, Peng J, Lian L (2023). Advanced lung cancer inflammation index predicts the outcomes of patients with non-metastatic gastric cancer after radical surgical resection. J Gastrointest Oncol.

[29] Yin C, Toiyama Y, Okugawa Y, Omura Y, Kusunoki Y, Kusunoki K, Imaoka Y, Yasuda H, Ohi M, Kusunoki M (2021). Clinical significance of advanced lung cancer inflammation index, a nutritional and inflammation index, in gastric cancer patients after surgical resection: a propensity score matching analysis. Clin Nutr.

[30] Jafri SH, Shi R, Mills G (2013). Advance lung cancer inflammation index (ALI) at diagnosis is a prognostic marker in patients with metastatic non-small cell lung cancer (NSCLC): a retrospective review. BMC Cancer.

[31] Wei C, Yu Z, Wang G, Zhou Y, Tian L (2020). Low pretreatment albumin-to-globulin ratio predicts poor prognosis in gastric Cancer: insight from a Meta-analysis. Front Oncol.

[32] Mao MJ, Wei XL, Sheng H, Wang XP, Li XH, Liu YJ, Xing S, Huang Q, Dai SQ, Liu WL (2017). Clinical significance of preoperative albumin and globulin ratio in patients with gastric Cancer undergoing treatment. Biomed Res Int.

[33] Zhang Y, Zhu JY, Zhou LN, Tang M, Chen MB, Tao M (2020). Predicting the prognosis of gastric Cancer by Albumin/Globulin ratio and the Prognostic Nutritional Index. Nutr Cancer.

[34] Xue F, Lin F, Yin M, Feng N, Zhang X, Cui YG, Yi YP, Kong XY, Chen X, Liu WZ (2017). Preoperative albumin/globulin ratio is a potential prognosis predicting biomarker in patients with resectable gastric cancer. Turk J Gastroenterol.

[35] Hirahara T, Arigami T, Yanagita S, Matsushita D, Uchikado Y, Kita Y, Mori S, Sasaki K, Omoto I, Kurahara H (2019). Combined neutrophil-lymphocyte ratio and platelet-lymphocyte ratio predicts chemotherapy response and prognosis in patients with advanced gastric cancer. BMC Cancer.

[36] Cupp MA, Cariolou M, Tzoulaki I, Aune D, Evangelou E, Berlanga-Taylor AJ (2020). Neutrophil to lymphocyte ratio and cancer prognosis: an umbrella review of systematic reviews and meta-analyses of observational studies. BMC Med.

[37] Nogueiro J, Santos-Sousa H, Pereira A, Devezas V, Fernandes C, Sousa F, Fonseca T, Barbosa E, Barbosa JA (2022). The impact of the prognostic nutritional index (PNI) in gastric cancer. Langenbecks Arch Surg.

[38] Mantovani A, Allavena P, Sica A, Balkwill F (2008). Cancer-related inflammation. Nature.

[39] Labelle M, Begum S, Hynes RO (2011). Direct signaling between platelets and cancer cells induces an epithelial-mesenchymal-like transition and promotes metastasis. Cancer Cell.

[40] Shi H, Jiang Y, Cao H, Zhu H, Chen B, Ji W (2018). Nomogram based on systemic Immune-inflammation index to predict overall survival in gastric Cancer patients. Dis Markers.

[41] Wang X, Mao M, Zhu S, Xing S, Song Y, Zhang L, Chi P (2020). A Novel Nomogram Integrated with inflammation-based factors to predict the prognosis of gastric Cancer patients. Adv Ther.

[42] Wang PX, Wang HJ, Liu JH, Qiu GL, Lu J, Fan L, Liao XH, Che XM (2021). A nomogram combining plasma fibrinogen and systemic immune–inflammation index predicts survival in patients with resectable gastric cancer. Sci Rep.

[43] Rihawi K, Ricci AD, Rizzo A, Brocchi S, Marasco G, Pastore LV, Llimpe FLR, Golfieri R, Renzulli M. Tumor-Associated macrophages and Inflammatory Microenvironment in Gastric Cancer: Novel Translational implications. Int J Mol Sci 2021, 22(8). 10.3390/ijms22083805 PMC8067563 33916915

[44] Liang S, Wei C, Tang L, Gao J, Yan W, Wu J, Long Z, Wang Y (2022). Clinical value and application of a novel nomogram containing inflammatory, nutritional and clinical markers in predicting overall survival of stage II/III gastric cancer patients after radical resection: a bi-centered retrospective study of 2,443 patients. Am J Transl Res.

[45] Zhang J, Ding Y, Wang W, Lu Y, Wang H, Wang H, Teng L (2020). Combining the Fibrinogen/Albumin ratio and systemic inflammation response index predicts survival in Resectable Gastric Cancer. Gastroenterol Res Pract.

[46] Bang CS, Ahn JY, Kim JH, Kim YI, Choi IJ, Shin WG (2021). Establishing machine learning models to predict curative resection in early gastric Cancer with undifferentiated histology: development and usability study. J Med Internet Res.

[47] Zhou C, Hu J, Wang Y, Ji MH, Tong J, Yang JJ, Xia H (2021). A machine learning-based predictor for the identification of the recurrence of patients with gastric cancer after operation. Sci Rep.

[48] Zhou C, Wang Y, Ji MH, Tong J, Yang JJ, Xia H (2020). Predicting Peritoneal Metastasis of Gastric Cancer patients based on machine learning. Cancer Control.

[49] Zhou CM, Wang Y, Yang JJ, Zhu Y (2023). Predicting postoperative gastric cancer prognosis based on inflammatory factors and machine learning technology. BMC Med Inf Decis Mak.

[50] Turkki R, Byckhov D, Lundin M, Isola J, Nordling S, Kovanen PE, Verrill C, von Smitten K, Joensuu H, Lundin J (2019). Breast cancer outcome prediction with tumour tissue images and machine learning. Breast Cancer Res Treat.

[51] Wang L, Su M, Zhang M, Zhao H, Wang H, Xing J, Guo C, Zhou D, Xue W, Lu H (2021). Accurate prediction of prognosis by integrating clinical and molecular characteristics in Colon cancer. Front Cell Dev Biol.

