# Supplementary material for: Development and validation of an inflammatory biomarkers model to predict gastric cancer prognosis: a multi-center cohort study in China
Source: BMC Cancer. 2024 Jun 10;24:711. doi: 10.1186/s12885-024-12483-4 (PMC11163779; doi:10.1186/s12885-024-12483-4)
Supplement: Supplementary file 1 — Supplementary Material 1 [file 12885_2024_12483_MOESM1_ESM.docx]

Supplemental Material

# Supplemental Figures and Tables

## Supplemental Tables

**Supplemental Table 1.** Inclusion and exclusion criteria

| **Inclusion criteria** | **Exclusion criteria** |
| --- | --- |
| age at least 18 years old; | 1) with more than two types of cancers at the same time; |
| 2) pathologically diagnosed with gastric cancer; | 2) with missing follow-up information; |
| 3) conscious and able to answer questions independently; | 3) lack of detailed clinical and biochemical information, including baseline data such as tumor stage, family history, and blood biochemical test results such as albumin, globulin, neutrophil count, lymphocyte count. |
| hospitalized for anticancer treatment from January 2013 to November 2021. |  |

**Supplemental Table 2.** Procedures used to obtain the inflammatory markers information in the present study

| **Indicators** | **Definition or calculation formula** | |
| --- | --- | --- |
| BMI | weight (kg)/height^2^ (m^2^ ) | |
| ALI | BMI (kg/m^2^) × albumin(g/dl)/NLR (×10^9^) | |
| SII | platelet count (×10^9^) × neutrophil count (×10^9^)/lymphocyte count (×10^9^) | |
| PLR | platelet count (×10^9^)/lymphocyte count (×10^9^) | |
| NLR | neutrophil count (×10^9^)/ lymphocyte count (×10^9^) | |
| PNI | albumin (g/L) + 5×lymphocyte count (×10^9^) | |
| CAR | C-reactive protein (mg/ L)/ albumin (g/ L) | |
| GLR | glucose (mmol/L)/ lymphocyte count (×10^9^) | |
| LCR | 10,000×lymphocyte count (×10^9^)/CRP (mg/L) | |
| AGR | albumin (g/L)/globulin (g/L) | |
| PGR | Prealbumin(mg/L)/globulin (g/L) | |
| Nutritional Risk Index | 1.519× albumin (g/L) + 41.7× current weight/ IBW | |
| mGPS |  | |
| Score 0 | CRP (≤10 mg/L) and albumin (≥35 g/L) | |
| Score 0 | CRP (≤10 mg/L) and albumin (<35 g/L) | |
| Score 1 | CRP (>10 mg/L) and albumin (≥35 g/L) | |
| Score 2 | CRP (>10 mg/L) and albumin (<35 g/L) | |
| LCS |  | |
| Score 0 | lymphocyte count ≥1×10^9^/L and CRP ≤3.0 mg/L | |
| Score 1 | lymphocyte count <1×10^9^/L and CRP ≤3.0 mg/L | |
| Score 1 | lymphocyte count ≥1×10^9^/L and CRP >3.0 mg/L | |
| Score 2 | lymphocyte count <1×10^9^/L and CRP >3.0 mg/L | |
| CONUT score |  | |
| Serum albumin (g/dL) | | albumin score |
| ≥35 | | 1 |
| 30-34.9 | | 2 |
| 25-29.9 | | 4 |
| <25 | | 6 |
| Total lymphocyte (count/mm^3^) | | TLC score |
| ≥1600 | | 0 |
| 1200-1599 | | 1 |
| 800-1199 | | 2 |
| <800 | | 3 |
| Total cholesterol (mg/dL) | | T-cho score |
| ≥180 | | 0 |
| 140-180 | | 1 |
| 100-139 | | 2 |
| <100 | | 3 |
| CONUT score (total) | | albumin score+ TLC score+ T-cho score |

**Supplemental Table 3.** Procedures used to obtain the inflammatory markers information in the present study

| **Model** | **Key parameters** | **Packages used** |
| --- | --- | --- |
| Decision tree(Decision tree (conditional inference tree) | min split=20, max depth=30, min bucket=7 | ctree |
| Random forest (traditional) | n trees=500, n variables=the square root of the input variables | randomForest |
| Adaptive boost machine | n trees=50, max depth=30, min split=20, complexity parameter=0.01, cross validation=10 fold, iteration=50 | ada |
| Supportive vector machine | Kernel=Radial Basis (rbfdot) | ksvm |
| Logistic regression | - | glm |
| Neural network | hidden layer nodes=10 | nnet |

**Supplemental Table 4.**  Results of feature selection

| **Ranking** | **Model, metric and variables selected, top 30% (n=13) descending** | | | | | |
| --- | --- | --- | --- | --- | --- | --- |
|  | **DT** | **RF** | **ADA** | **SVM** | **LR** | **NNET** |
| 1 | TNM | TNM | TNM | TNM | TNM | CRP |
| 2 | PGR | PGR | PGR | PGR | EORTCQLQC30 | SII |
| 3 | PALB | glucose | PALB | PALB | ALI | GLR |
| 4 | AGR | age | AGR | AGR | GLR | PLR |
| 5 | LCR | PALB | LCR | LCR | therapy | glucose |
| 6 | CAR | TCLmmol | CAR | CAR | SII | TNM |
| 7 | CRP | RBC | CRP | CRP | PLT | PG.SGA |
| 8 | ALI | hemoglobin | ALI | ALI | NLR | LYM |
| 9 | NLR | PLT | NLR | NLR | smoking | LCS |
| 10 | SII | LCR | SII | SII | age | ALI |
| 11 | NEU | AGR | NEU | NEU | LCS | hypertension |
| 12 | ALB | NEU | ALB | ALB | KPS | PALB |
| 13 | PNI | GLB | PNI | PNI | PLR | GLB |

**Supplemental Table 5.** Final results of feature selection

| **Ranking** | **Model, metric and variables selected, top 30% (n=13) descending** | | | | | |
| --- | --- | --- | --- | --- | --- | --- |
|  | **DT** | **RF** | **ADA** | **SVM** | **LR** | **NNET** |
| 1 | TNM | TNM | TNM | TNM | TNM | CRP |
| 2 | PGR | PGR | PGR | PGR | EORTCQLQC30 | SII |
| 3 | PALB | glucose | PALB | PALB | ALI | glucose |
| 4 | AGR | age | AGR | AGR | therapy | TNM |
| 5 | CAR | PALB | CAR | CAR | SII | PG.SGA |
| 6 | CRP | TCLmmol | CRP | CRP | PLT | LYM |
| 7 | ALI | RBC | ALI | ALI | NLR | ALI |
| 8 | NLR | hemoglobin | NLR | NLR | smoking | hypertension |
| 9 | SII | PLT | SII | SII | age | PALB |
| 10 | NEU | AGR | NEU | NEU | KPS | GLB |
| 11 | ALB | NEU | ALB | ALB | NRS2002 | NRS2002 |
| 12 | PNI | GLB | PNI | PNI | LYM | EORTCQLQC30 |
| 13 | Nutritional Risk Index | PNI | Nutritional Risk Index | Nutritional Risk Index | history | NLR |

**Supplemental** **Table 6.** Results of the Cox analysis

| **Characteristics** | **HR (95%CI)** | **P** |
| --- | --- | --- |
| TNM |  |  |
| Ⅰ | Reference (HR = 1) |  |
| Ⅱ | 2.544 (1.278-5.066) | 0.008 |
| Ⅲ | 3.931 (2.057-7.514) | <0.001 |
| Ⅳ | 8.696 (4.479-16.882) | <0.001 |
| PGR | 1.045 (0.892-1.225) | 0.584 |
| PALB | 0.998 (0.991-1.004) | 0.408 |
| AGR | 0.510 (0.254-1.024) | 0.058 |
| CAR | 1.004 (0.557-1.812) | 0.989 |
| CRP | 1.005 (0.986-1.023) | 0.628 |
| ALI | 0.993 (0.988-0.998) | 0.009 |
| NLR | 0.956 (0.897-1.020) | 0.172 |
| SII | 1.000 (1.000-1.000) | 0.272 |
| NEU | 0.983 (0.941-1.026) | 0.421 |
| ALB | 0.981 (0.944-1.020) | 0.330 |
| PNI | 1.020 (0.992-1.050) | 0.165 |
| Nutritional Risk Index | 1.003 (0.983-1.024) | 0.763 |

Notes: Risk score of the Cox model: 0.9337 × tumor stage II (yes = 1, no = 0) + 1.3689 × tumor stage III (yes = 1, no = 0) + 2.1629 × tumor stage IV (yes = 1, no = 0) + 0.0443 × PGR (kg/m2) − 0.0026 × PALB (mg/L) − 0.6734 × AGR + 0.0040 × CAR + 0.0045 × CRP (mg/L) − 0.0068 × ALI − 0.0447 × NLR + 0.0001 × SII − 0.0176 × NEU (×109/L) − 0.0191 × ALB (g/L) + 0.0200 × PNI + 0.0032 × Nutritional Risk Index.

**Supplemental Table 7.** The univariate and multivariate analysis of risk score in total patients and different sets

| Variables | OS (model 0) | |  | OS (model 1) | |  | OS (model 2) | |
| --- | --- | --- | --- | --- | --- | --- | --- | --- |
|  | Crude HR (95%CI) | Crude P |  | Adjusted HR (95%CI) | Adjusted P |  | Adjusted HR (95%CI) | Adjusted P |
| Total patients |  |  |  |  |  |  |  |  |
| Risk Score | 2.494 (2.372-2.616) | <0.001 |  | 2.515 (2.392-2.638) | <0.001 |  | 2.158 (1.933-2.383) | <0.001 |
| Low risk group | Reference (HR = 1) |  |  | Reference (HR = 1) |  |  | Reference (HR = 1) |  |
| High risk group | 4.152 (3.946-4.358) | <0.001 |  | 4.150 (3.944-4.356) | <0.001 |  | 3.072 (2.672-3.472) | <0.001 |
| Training cohort |  |  |  |  |  |  |  |  |
| Risk Score | 2.718 (2.553-2.883) | <0.001 |  | 2.730 (2.563-2.897) | <0.001 |  | 2.481 (2.150-2.812) | <0.001 |
| Low risk group | Reference (HR = 1) |  |  | Reference (HR = 1) |  |  | Reference (HR = 1) |  |
| High risk group | 4.046 (3.795-4.297) | <0.001 |  | 4.010 (3.755-4.265) | <0.001 |  | 3.131 (2.623-3.639) | <0.001 |
| Validation cohort |  |  |  |  |  |  |  |  |
| Risk Score | 2.224 (2.034-2.414) | <0.001 |  | 2.247 (2.053-2.441) | <0.001 |  | 2.088 (1.733-2.443) | <0.001 |
| Low risk group | Reference (HR = 1) |  |  | Reference (HR = 1) |  |  | Reference (HR = 1) |  |
| High risk group | 4.350 (3.987-4.713) | <0.001 |  | 4.413 (4.046-4.780) | <0.001 |  | 3.361 (2.685-4.037) | <0.001 |

Model 0: non-adjustment model.

Model 1:adjusted for age, gender, alcohol, history.

Model 2: adjusted for age, gender, TNM, BMI, smoking, alcohol, KPS, therapy, diabetes, hypertension, history, PG-SGA, NRS2002, EORTC QLQ-C30.

**Supplemental Table 8.** Sensitivity analysis of risk score in the training cohort, validation cohort and total patients

| Variables | OS (model 0) | |  | OS (model 1) | |  | OS (model 2) | |
| --- | --- | --- | --- | --- | --- | --- | --- | --- |
|  | Crude HR (95%CI) | Crude P |  | Adjusted HR (95%CI) | Adjusted P |  | Adjusted HR (95%CI) | Adjusted P |
| Total patients |  |  |  |  |  |  |  |  |
| Risk Score | 2.471 (2.342-2.600) | <0.001 |  | 2.485 (2.354-2.616) | <0.001 |  | 2.119 (1.876-2.362) | <0.001 |
| Low risk group | Reference (HR = 1) |  |  | Reference (HR = 1) |  |  | Reference (HR = 1) |  |
| High risk group | 4.060 (3.842-4.278) | <0.001 |  | 4.046 (3.826-4.266) | <0.001 |  | 2.905 (2.474-3.336) | <0.001 |
| Training cohort |  |  |  |  |  |  |  |  |
| Risk Score | 2.618 (2.444-2.792) | <0.001 |  | 2.610 (2.432-2.788) | <0.001 |  | 2.326 (1.971-2.681) | <0.001 |
| Low risk group | Reference (HR = 1) |  |  | Reference (HR = 1) |  |  | Reference (HR = 1) |  |
| High risk group | 3.770 (3.501-4.039) | <0.001 |  | 3.700 (3.430-3.970) | <0.001 |  | 2.575 (2.014-3.136) | <0.001 |
| Validation cohort |  |  |  |  |  |  |  |  |
| Risk Score | 2.288 (2.086-2.490) | <0.001 |  | 2.314 (2.110-2.518) | <0.001 |  | 2.289 (1.905-2.673) | <0.001 |
| Low risk group | Reference (HR = 1) |  |  | Reference (HR = 1) |  |  | Reference (HR = 1) |  |
| High risk group | 4.704 (4.318-5.090) | <0.001 |  | 4.805 (4.415-5.195) | <0.001 |  | 4.288 (3.573-5.003) | <0.001 |

Notes: * The sensitivity analysis was to exclude patients who died within 6 months.

Model 0: non-adjustment model.

Model 1:adjusted for age, gender, alcohol, history.

Model 2: adjusted for age, gender, TNM, BMI, smoking, alcohol, KPS, therapy, diabetes, hypertension, history, PG-SGA, NRS2002, EORTC QLQ-C30.

**Supplemental Table 9.** Sensitivity analysis of risk score in total patients

| Variables | OS (model 0) | |  | OS (model 1) | |  | OS (model 2) | |
| --- | --- | --- | --- | --- | --- | --- | --- | --- |
|  | Crude HR (95%CI) | Crude P |  | Adjusted HR (95%CI) | Adjusted P |  | Adjusted HR (95%CI) | Adjusted P |
| Total patients |  |  |  |  |  |  |  |  |
| Risk Score | 2.697 (2.328-3.126) | <0.001 |  | 2.721 (2.342-3.162) | <0.001 |  | 2.698 (1.826-3.985) | <0.001 |
| Low risk group | Reference (HR = 1) |  |  | Reference (HR = 1) |  |  | Reference (HR = 1) |  |
| High risk group | 3.681 (2.972-4.559) | <0.001 |  | 3.757 (3.019-4.676) | <0.001 |  | 1.432 (1.210-1.696) | <0.001 |

Model 0: non-adjustment model.

Model 1:adjusted for age, gender, alcohol, history.

Model 2: adjusted for age, gender, TNM, BMI, smoking, alcohol, KPS, therapy, diabetes, hypertension, history, PG-SGA, NRS2002, EORTC QLQ-C30.

## Supplemental Figures


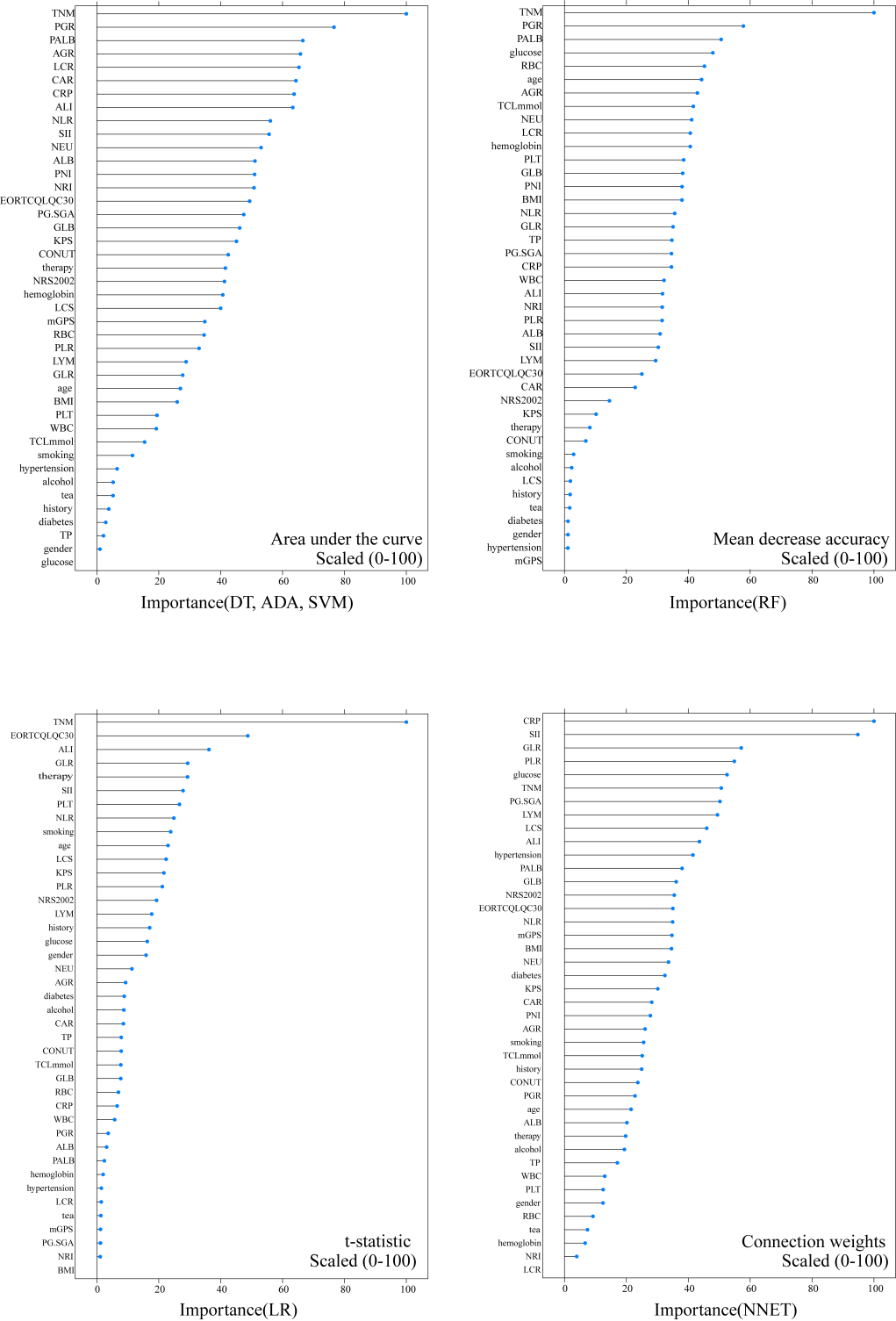


**Supplemental Figure 1.** The relative importance of the input variables in the full models.


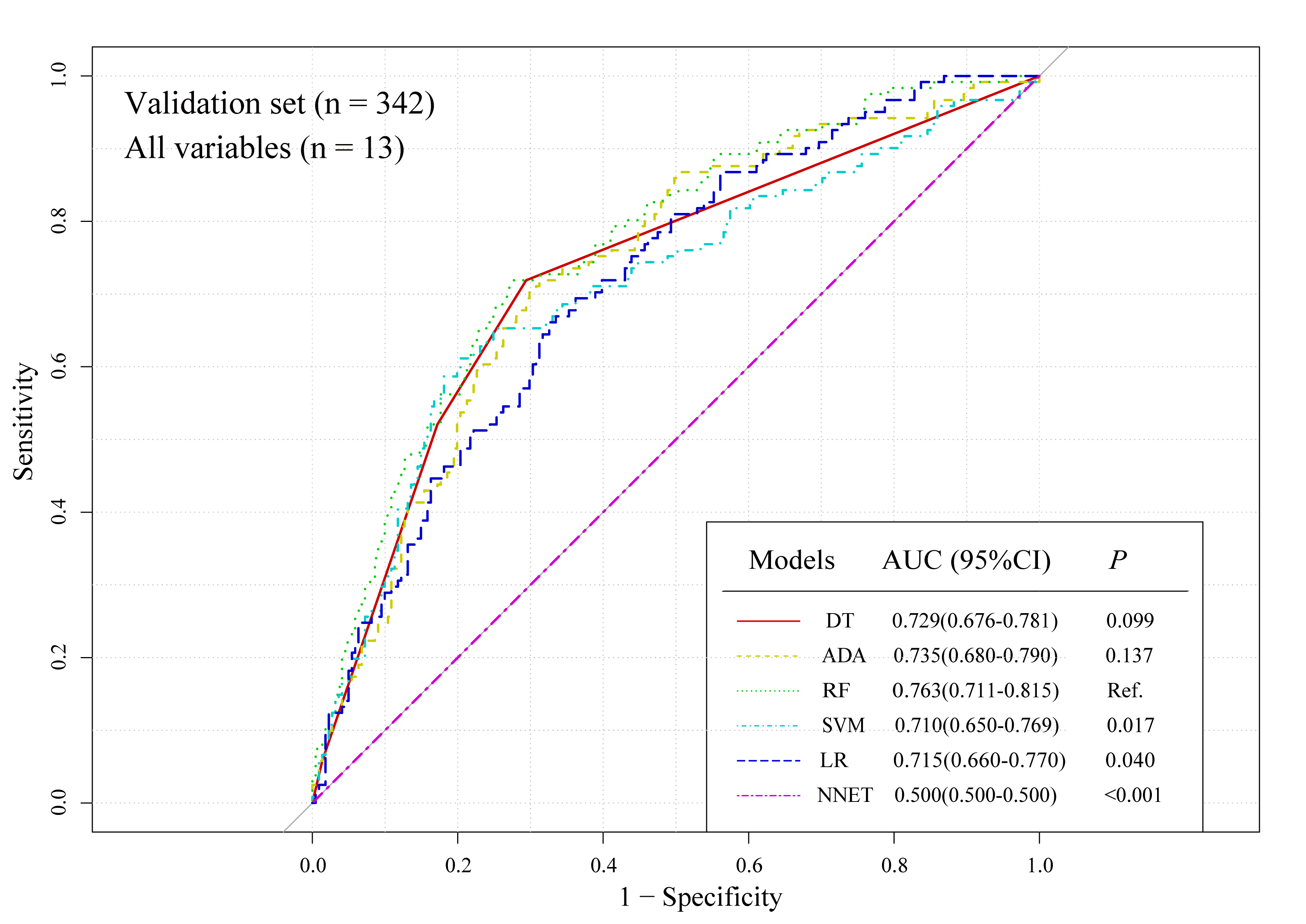


**Supplemental Figure 2.** Performance of the simplified ML models without PH Assumption after feature selection in validation data.

**
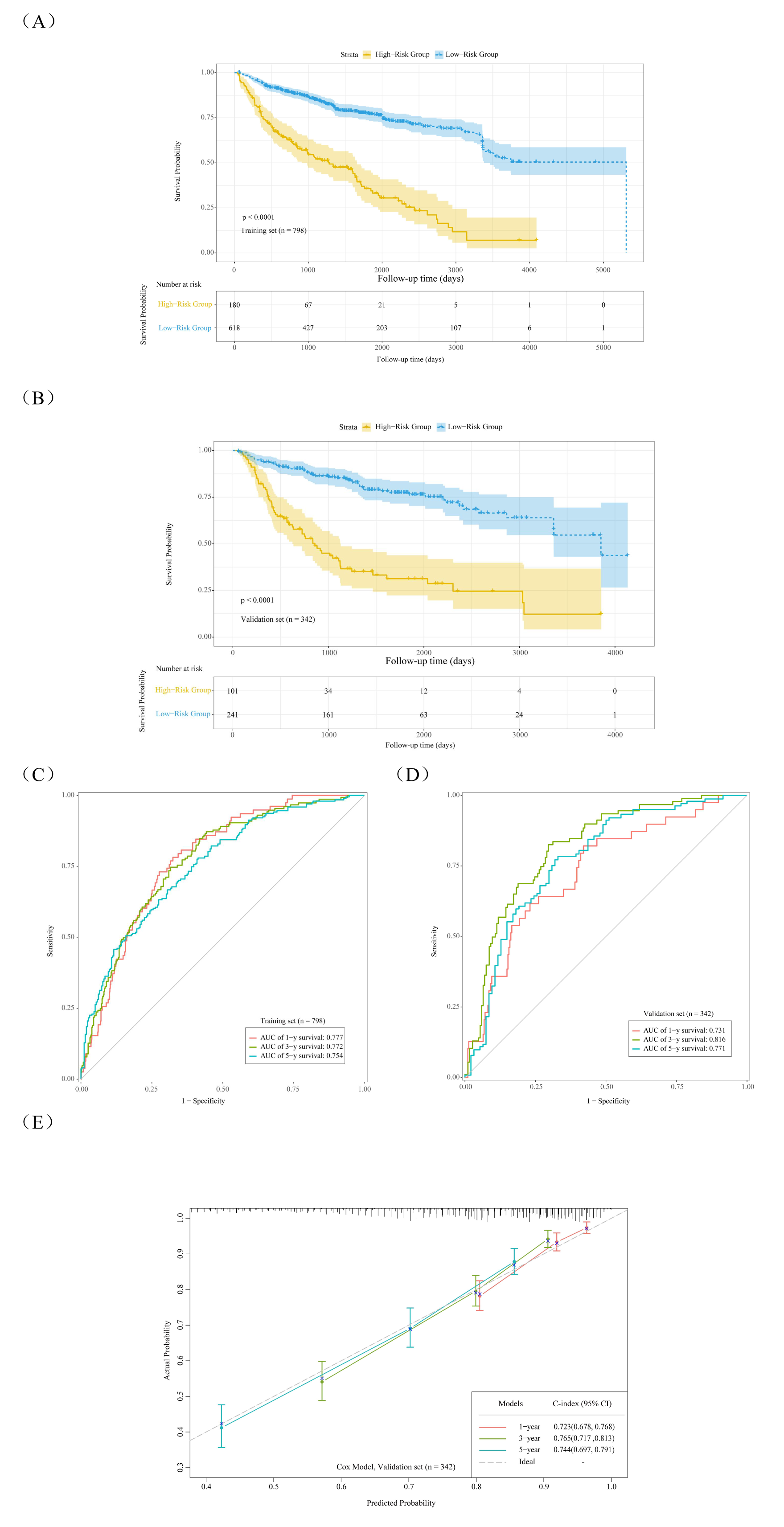
**

**Supplemental Figure 3.** Cox Model performance in the training and validation sets. (a) The Kaplan-Meier survival curves of Risk Score calculated by Cox Model in the training set. (b) The Kaplan-Meier survival curves of Risk Score calculated by Cox Model in the validation set. (c) Comparison of 1-, 3-, and 5-year prognostic ROC for Risk Score calculated by Cox Model in training cohort. (d) Comparison of 1-, 3-, and 5-year prognostic ROC for Risk Score calculated by Cox Model in the validation cohort. (e) Comparison of 1-, 3-, and 5-year calibration curves of the nomogram.


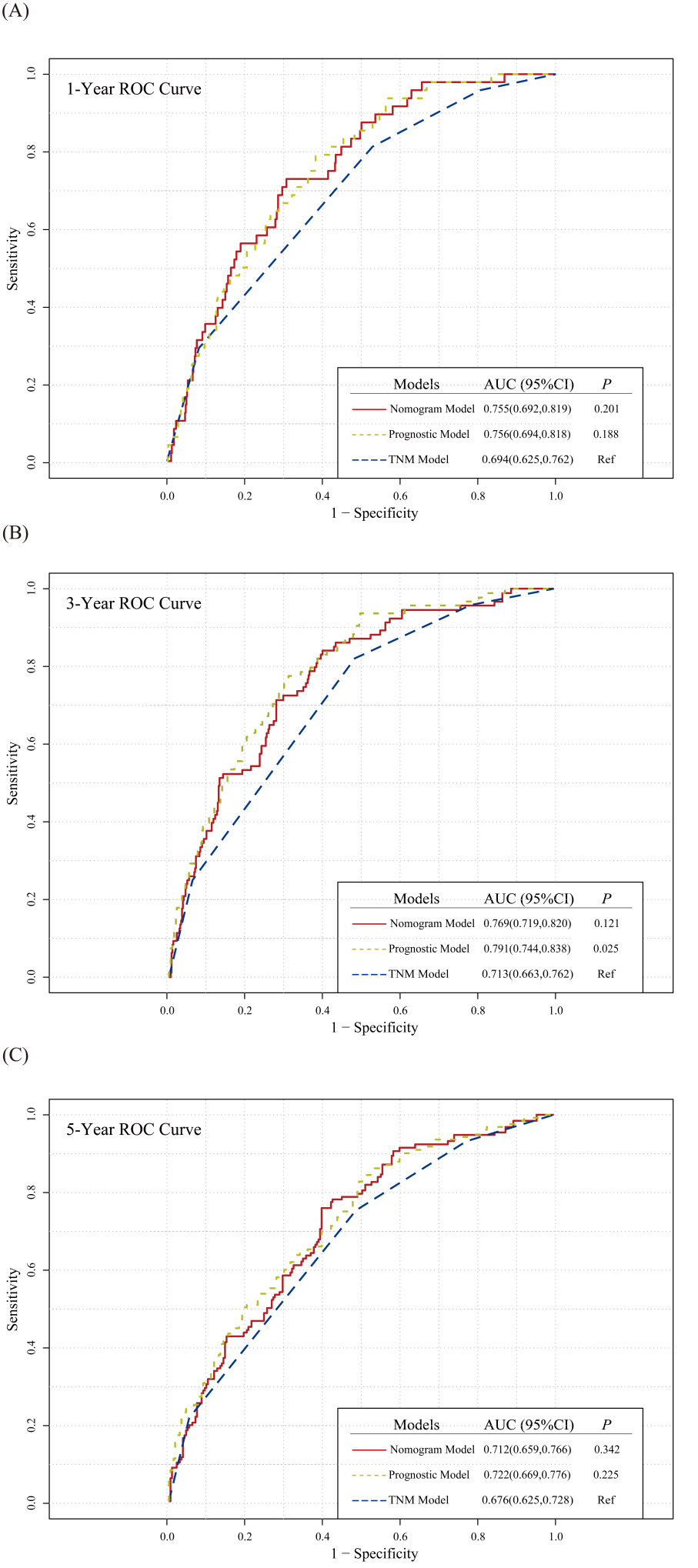


**Supplemental Figure 4.** Nomogram Model performance in patients undergoing surgical treatment (AUCs were compared using DeLong’s test). (a) Comparison of 1-year prognostic ROC for Risk Score calculated by Nomogram Model, Cox Model and TNM Model in patients undergoing surgical treatment. (b) Comparison of 3-year prognostic ROC for Risk Score calculated by Nomogram Model, Cox Model and TNM Model in patients undergoing surgical treatment. (c) Comparison of 5-year prognostic ROC for Risk Score calculated by Nomogram Model, Cox Model and TNM Model in patients undergoing surgical treatment.


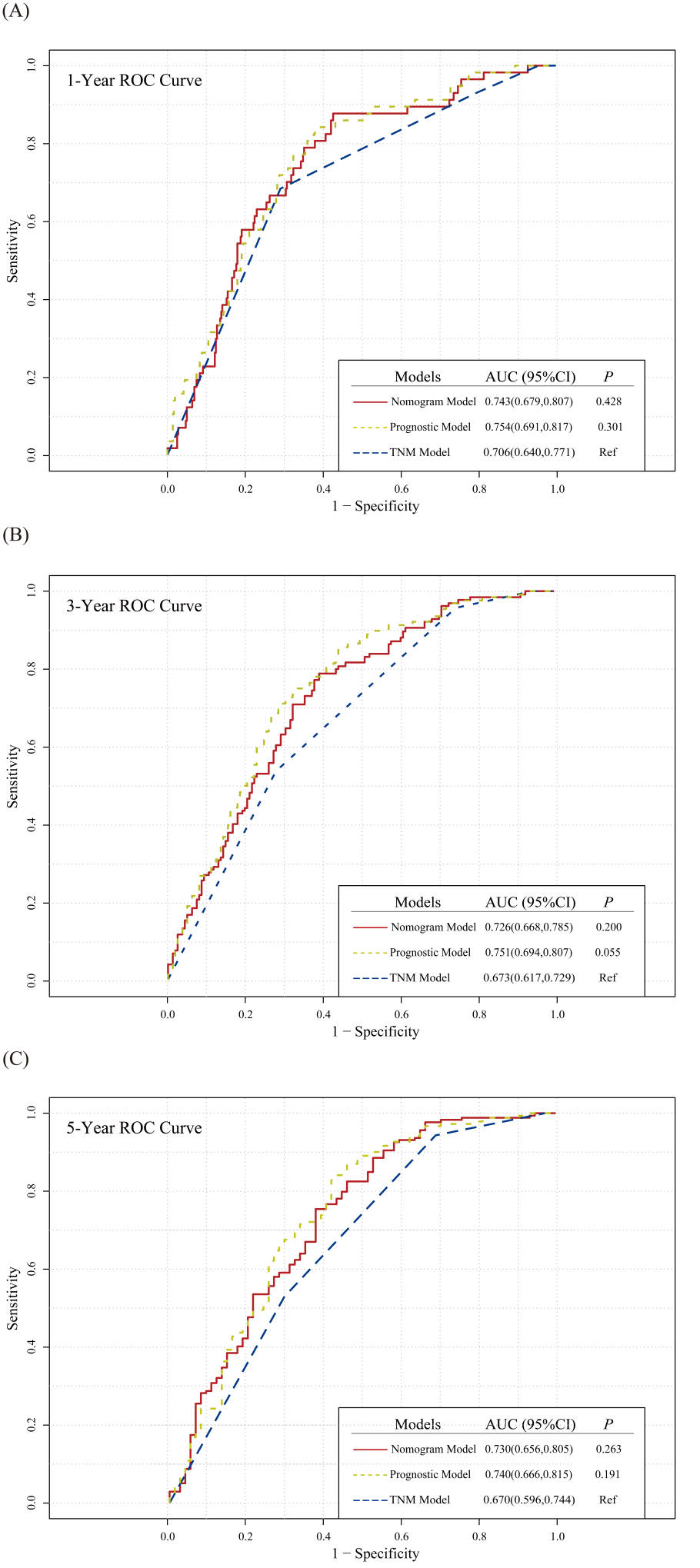


**Supplemental Figure 5.** Nomogram Model performance in patients receiving chemotherapy (AUCs were compared using DeLong’s test). (a) Comparison of 1-year prognostic ROC for Risk Score calculated by Nomogram Model, Cox Model and TNM Model in patients receiving chemotherapy. (b) Comparison of 3-year prognostic ROC for Risk Score calculated by Nomogram Model, Cox Model and TNM Model in patients receiving chemotherapy. (c) Comparison of 5-year prognostic ROC for Risk Score calculated by Nomogram Model, Cox Model and TNM Model in patients receiving chemotherapy.


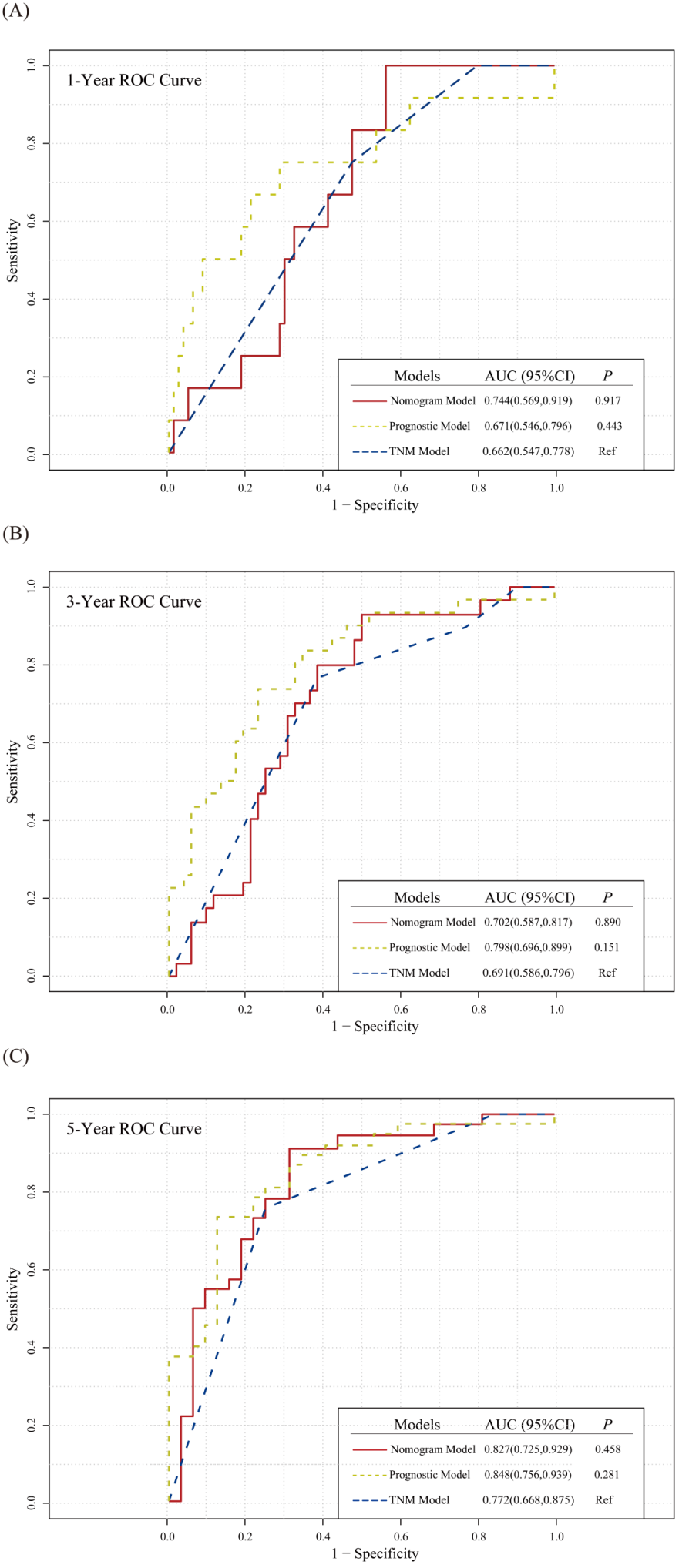


**Supplemental Figure 6.** Nomogram Model performance in patients receiving other treatments (AUCs were compared using DeLong’s test). (a) Comparison of 1-year prognostic ROC for Risk Score calculated by Nomogram Model, Cox Model and TNM Model in patients receiving other treatments. (b) Comparison of 3-year prognostic ROC for Risk Score calculated by Nomogram Model, Cox Model and TNM Model in patients receiving other treatments. (c) Comparison of 5-year prognostic ROC for Risk Score calculated by Nomogram Model, Cox Model and TNM Model in patients receiving other treatments.
